# Assistive Robots for Healthcare and Human–Robot Interaction

**DOI:** 10.3390/s23041883

**Published:** 2023-02-08

**Authors:** Grazia D’Onofrio, Daniele Sancarlo

**Affiliations:** 1 Clinical Psychology Service, Health Department, Fondazione IRCCS Casa Sollievo della Sofferenza, San Giovanni Rotondo, 71013 Foggia, Italy; 2 Complex Unit of Geriatrics, Department of Medical Sciences, Fondazione IRCCS Casa Sollievo della Sofferenza, San Giovanni Rotondo, 71013 Foggia, Italy

Assistive robots are still mostly prototypes that only remotely recall human interactive dynamics. Researchers are working to create, in record time, a being that can assist us, encourage us, teach us, and support precision and heavy work activities, becoming—on the basis of its purpose of use—the possibly perfect interactive partner. However, to do this, it will be necessary to go far beyond the already advanced robotic implementations that make non-verbal communication the pivot on which the human–robot interaction (HRI) is based.

The study of the HRI is a relatively new research area. The field of investigation is broad and diverse, and both hardware and software design processes face interesting and open challenges. Presently, various fields of application and the use of assistive robots are being examined by the scientific community. For these reasons, the development of the HRI makes use of the contribution of various disciplines, ranging from those with a more mathematical-engineering imprint to the more humanistic sciences. The theme is then opened up to numerous issues of an ethical and legal nature, which are also still being explored. Thanks to the entry on the market of relatively affordable models, assistive robots are gradually expanding in society and can provide initial support to human activities, albeit still in an experimental form and in structured places.

By definition, the interaction implicates the communication. In light of this assumption, research in the HRI field is increasingly focused on the development of robots equipped with intelligent communicative abilities, in particular speech-based natural language conversational abilities. These efforts directly relate to the research area of computational linguistics, generally defined as “the subfield of computer science concerned with using computational techniques to learn, understand, and produce human language content”. The advances and results in computational linguistics provide a foundational background for the development of so-called Spoken Dialogue Systems, i.e., computer systems designed to interact with humans using spoken natural language. The ability to communicate using natural language is a fundamental requirement for a robot that interacts with a human being. Then, spoken dialogue is generally considered as the most natural way for a social human–robot interaction.

The main topic of this Special Issues has been to advance the novel technologies applied in healthcare processes that have shown exceptional promise in models of an HRI. The first important question concerns the modalities needed to sense the emotional state of people by the robot. Second, there is the problem of modeling the interaction between human and robot, not only on a haptic level, but also on an emotional level.

The Special Issue is a collection of papers targeting an audience of practicing researchers, academics, and other scientists from Canada, France, Greece, Italy, Japan, Korea, Poland, Saudi Arabia, Spain, Taiwan, and the USA. Its contents were written by multiple authors and edited by experts in clinical and researcher fields.

They contributed to increasing the knowledge on assistive robots and the HRI considering, as enablers, to support the process of care giving, potentially enhancing patients’ well-being and decreasing the caregiver workload.

In the first study, Kim and colleagues [1] discussed studies on care robots and the human-centered artificial intelligence framework, presented an ethical design for the sensing services of care robots, and reported the development of a care robot for frail older adult users.

In the second study, Aygun et al. [2] analyzed and modeled data from a multi-modal simulated driving study specifically designed to evaluate different levels of cognitive workload induced by various secondary tasks, such as dialogue interactions and braking events, in addition to the primary driving task. They performed statistical analyses of various physiological signals including eye gaze, electroencephalography, and arterial blood pressure from the healthy volunteers and utilized several machine learning methodologies, including k-nearest neighbor, naive Bayes, random forest, support-vector machines, and neural network-based models to infer human cognitive workload levels.

In the third study, Michel et al. [3] presented a prototype robot capable of supporting a surgeon during otological surgery. On the basis of the observation that patients may move or wake up during an operation, and that the surgeon must regularly clean the endoscope optics, new robot architecture was presented.

In the successive work, Julia Arias-Rodríguez and colleagues [4] discussed the feasibility and validation of a microwave antenna-based imaging system for intra-operative surgical navigation. The authors reported that the experimental assessment of the proposed system showed accuracies and errors consistent with other approaches with other technologies found in the literature, thus highlighting the interest for further studies.

In the fifth study, Grazia D’Onofrio et al. [5] identified if traditional machine learning algorithms could be used to assess every users’ emotions separately, to relate emotion recognizing in two robotic modalities, a static or motion robot, and to evaluate the acceptability and usability of an assistive robot from an end-user point of view. The authors reported that the random forest algorithm’s performance was better in terms of accuracy and execution time than k-nearest neighbor’s algorithm, and the robot was not a disturbing factor in the arousal of emotions.

Slawomir Tobis et al.’s [6] study focused on technology acceptance. They posed the question of whether there was a possibility of interacting with the technology and if this had an impact on the scores awarded by the respondents in various domains of the needs and requirements for social robots to be deployed in the care of older adults. The authors concluded that pre-implementation studies and assessments should include the possibility of interacting with the robot to provide its future users with a clear idea of the technology and facilitate the necessary customizations of the machine.

In the seventh study, Kazuyuki Matsumoto and colleagues [7] focused on interview dialogue systems, proposing a method based on a multi-task learning neural network that uses embedded representations of sentences to understand the context of the text and utilizes the intention of an utterance as a feature.

In the successive manuscript, Chris Lytridis et al. [8] presented novel tools for the analysis of human behavior data regarding robot-assisted special education for children with autism spectrum disorder (ASD). The authors included an understanding of human behavior in response to an array of robot actions and an improved intervention design based on suitable mathematical instruments.

Grazia D’Onofrio and colleagues [9] determined the needs and preferences of older people and their caregivers for improving healthy and active aging and guiding the technological development of a technological system. Additionally, these authors highlighted the importance of pre-implementation studies in order to improve the acceptance of the technological systems by end-users.

Afterwards, Hsiao-Kuan Wu et al. [10] showed that the robot can follow the user in the designated position while the user performs forward, backward, and lateral movements, turning and walking along a curve.

Nan Liang and Goldie Nejat [11] presented the first comprehensive investigation and meta-analysis of two types of robotic presence to determine how they influence the HRI outcomes and impact user tasks.

In the final manuscript, Amal Alabdulkareem and colleagues [12] proposed a systematic review and asserted that robot-assisted therapy is a promising field of application for intelligent social robots, especially to support children with ASD in achieving their therapeutic and educational objectives (social and emotional development, communication and interaction development, cognitive development, motor development, sensory development, and areas other than developmental ones).

In light of this Special Issue, as the area of social robotics and HRI grows, public demonstrations have the potential to provide insights into the robot and system effectiveness in public settings and the reactions of the people. One of the challenges is that, although modeling the dynamics of expressions and emotions has been extensively studied in the literature, how to model personality in a time-continuous manner has been an open problem.
